# Novel therapies emerging in oncology to target the TGF-β pathway

**DOI:** 10.1186/s13045-021-01053-x

**Published:** 2021-04-06

**Authors:** Byung-Gyu Kim, Ehsan Malek, Sung Hee Choi, James J. Ignatz-Hoover, James J. Driscoll

**Affiliations:** 1 Division of Hematology and Oncology, Department of Medicine, Case Western Reserve University School of Medicine, Cleveland, OH USA; 2 Case Comprehensive Cancer Center, Case Western Reserve University, Cleveland, OH USA; 3 Adult Hematologic Malignancies and Stem Cell Transplant Section, Seidman Cancer Center, University Hospitals Cleveland Medical Center, Cleveland, OH USA; 4 Department of Pediatrics, Case Western Reserve University, Cleveland, OH USA

**Keywords:** Immunosuppression, Ligand traps, Small molecule inhibitors, TGF-β receptor antagonists, Vactosertib

## Abstract

The TGF-β signaling pathway governs key cellular processes under physiologic conditions and is deregulated in many pathologies, including cancer. TGF-β is a multifunctional cytokine that acts in a cell- and context-dependent manner as a tumor promoter or tumor suppressor. As a tumor promoter, the TGF-β pathway enhances cell proliferation, migratory invasion, metastatic spread within the tumor microenvironment and suppresses immunosurveillance. Collectively, the pleiotropic nature of TGF-β signaling contributes to drug resistance, tumor escape and undermines clinical response to therapy. Based upon a wealth of preclinical studies, the TGF-β pathway has been pharmacologically targeted using small molecule inhibitors, TGF-β-directed chimeric monoclonal antibodies, ligand traps, antisense oligonucleotides and vaccines that have been now evaluated in clinical trials. Here, we have assessed the safety and efficacy of TGF-β pathway antagonists from multiple drug classes that have been evaluated in completed and ongoing trials. We highlight Vactosertib, a highly potent small molecule TGF-β type 1 receptor kinase inhibitor that is well-tolerated with an acceptable safety profile that has shown efficacy against multiple types of cancer. The TGF-β ligand traps Bintrafusp alfa (a bifunctional conjugate that binds TGF-β and PD-L1), AVID200 (a computationally designed trap of TGF-β receptor ectodomains fused to an Fc domain) and Luspatercept (a recombinant fusion that links the activin receptor IIb to IgG) offer new ways to fight difficult-to-treat cancers. While TGF-β pathway antagonists are rapidly emerging as highly promising, safe and effective anticancer agents, significant challenges remain. Minimizing the unintentional inhibition of tumor-suppressing activity and inflammatory effects with the desired restraint on tumor-promoting activities has impeded the clinical development of TGF-β pathway antagonists. A better understanding of the mechanistic details of the TGF-β pathway should lead to more effective TGF-β antagonists and uncover biomarkers that better stratify patient selection, improve patient responses and further the clinical development of TGF-β antagonists.

## Background

The transforming growth factor-β (TGF-β) signaling pathway regulates a multitude of key processes including cellular growth, differentiation, apoptosis, motility, invasion, extracellular matrix production, angiogenesis, immune responses [1, 2]. Recent advances in understanding the molecular details of the TGF-β signaling cascade, and crosstalk with other pathways, have led to a more coherent roadmap of the programs governed by TGF-β [3–5]. However, difficulty in defining the effect of TGF-β stems from the context-dependent nature of the cytokines effect on different cell types. Cell fate decisions are not determined simply by the available nutrients in the surrounding environment but controlled by a network of communication signals.

TGF-β1, 2 and 3 are evolutionarily conserved, secreted, isoforms that are encoded as large protein precursors with 70–80% homology that govern fundamental aspects of cellular behavior [6, 7]. TGF-β is synthesized in a latent form that must be activated to allow for engagement of a tetrameric receptor complex composed of TGF-β receptors I and II (TGF-βRI, TGF-βRII). Activated TGF-β complexes, with additional factors, form a serine/threonine kinase complex that binds to TGF-β receptors composed of both type I and type II receptor subunits. After the binding of TGF-β, the type 2 receptor kinase (TGF-βRII ) promotes heterotetramerization and phosphorylation that activates the type 1 receptor kinase (TGF-β RI), followed by phosphorylation of Smad2/Smad3 triggering a signaling cascade.

**Fig. 1:**
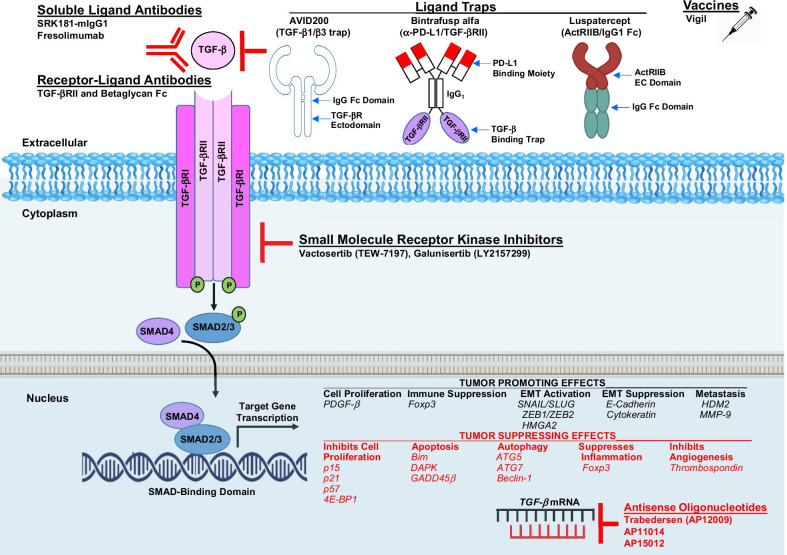
Agents in Development to Target the TGF-β Pathway in Oncology. Shown are noteworthy antagonists that target the TGF-β pathway and have recently been evaluated in clinical trials or are in clinical development. As indicated, many steps within the TGF-β pathway have been targeted therapeutically. Key tumor-promoting and tumor-suppressing genes transcriptionally regulated by the TGF-β pathwat are indicated

Activation of the TGF-β signaling pathway can elicit either tumor-suppressing or tumor-promoting effects in a cell- and context-dependent manner (Fig. 1). In normal tissues, these effects maintain homeostasis and prevent the early stages of tumor formation [8]. In healthy cells, TGF-β halts the cell cycle at G1 to reduce proliferation, induce differentiation and may promote apoptosis [4, 9, 10]. Activated Smad proteins associate with Smad4 and translocate to the nucleus where they recruit additional transcription factors, DNA-binding transcription factors, co-repressors, co-activators and chromatin remodeling factors. Differential expression of these factors may be responsible for the cell-type and context-dependent response to TGF-β. Nuclear localized SMAD2/3/4 complex induces tumor-promoting effects through cell proliferation (*PDGF*-β*),* immune suppression (*Foxp3),* EMT activation (*SNAIL/SLUG, ZEB1/ZEB2, HMGA2)*, EMT suppression *(E-Cadherin, Cytokeratin)* and metastasis (*HDM2, MMP-9)*. Tumor-suppressing effects inhibit cell proliferation (*p15, p21, p57, 4E-BP1*), apoptosis (*Bim, DAPK, GADD45*β), and autophagy (*ATG5, ATG7, Beclin-1*), suppress inflammation (*Foxp3*) and block angiogenesis (*Thrombospondin*).

In cancer cells, the TGF-β signaling pathway is deregulated or mutated, and TGF-β no longer controls proliferation. As a result of mutations or epigenetic modifications introduced during cancer progression, tumors can become resistant to the suppressive effects of TGF-β signaling [11]. TGF-β1 signaling is hyperactivated in breast cancer, which drives cancer progression and metastasis [12]. Novel regulators of SMAD7, including OTU domain-containing protein 1 (OTUD1) and ubiquitin-specific protease 26 (USP26), attenuate TGF-β1-mediated aggressive phenotypes in breast cancer. SMAD7 is an antagonist of the TGF-β1 signaling pathway forming stable complexes with TGF-βRI to inhibit R-SMAD phosphorylation and downstream signaling. In colon cancer, SMAD7 deletion predisposes to a favorable prognosis compared to patients with two copies of the gene, whereas patients with SMAD7 gene amplification exhibit significantly worse outcomes [13]. TGF-β1 and BMP-7 (bone morphogenetic protein-7), major members of the TGF-β family of receptor ligands, activate downstream signaling pathways and their respective target genes to counter-regulate biological outcomes. BMP7 deficiency accelerates tumorigenesis and negatively correlates with patient survival in prostate cancer [14]. Human prostatic carcinomas generally exhibit significantly lower levels of BMP7 transcripts compared to the normal-appearing gland; daily injections of recombinant BMP-7 protein reduced tumor burden and metastasis in mice transplanted with prostate cancer cell lines directly into the prostate or bone. Loss of BMP7 in glioblastoma, breast and prostate cancers, predisposes to enhanced EMT and acquisition of invasive/metastatic traits suggesting a protective role for BMP7 in cancer progression. Understanding the mechanisms by which the tumor-suppressing or tumor-promoting effects of TGF-β signaling can be regulated may have therapeutic potential for inhibiting the progression of several different types of human cancer.

In the tumor microenvironment (TME), TGF-β contributes to favorable tumor growth, invasion and metastatic spread. Advanced tumors produce excessive amounts of TGF-β which, in normal epithelial cells, is a potent growth inhibitor. However, in oncogenically activated cells, the homeostatic action of TGF-β is diverted along alternate pathways and elicits protective or tumor-suppressive effects during the early growth-sensitive stages of tumorigenesis. At late stages of malignancy, tumor progression is driven by TGF-β overload. The TME is a target of TGF-β action that stimulates tumor progression through pro-tumorigenic effects on tumor cells as well as vascular, immune and fibroblastic cells [4, 9, 10].

Epithelial to mesenchymal transition (EMT) is a multi-step, plastic and reversible process during which cells lose epithelial characteristics and gain properties of mesenchymal cells. The transition in cellular phenotype from EMT plays a critical role in tumorigenesis, tumor invasion and metastasis [15, 16], unless the mesenchymal phenotype becomes fixed by subsequent epigenetic changes or additional genetic mutations. The plasticity and reversibility of EMT in response to changing local TGF-β levels have clearly demonstrated in vitro and in vivo. TGF-β-induced EMT is an attractive druggable target since it is thought to drive a more stem cell-like phenotype which is critical for tumor progression, dissemination, homing and colony-initiating activity. Inhibiting TGF-β may reduce the stem cell-like tumor compartment which displays chemotherapeutic resistance.

## TGF-β effect on escape from immune surveillance

**Fig. 2:**
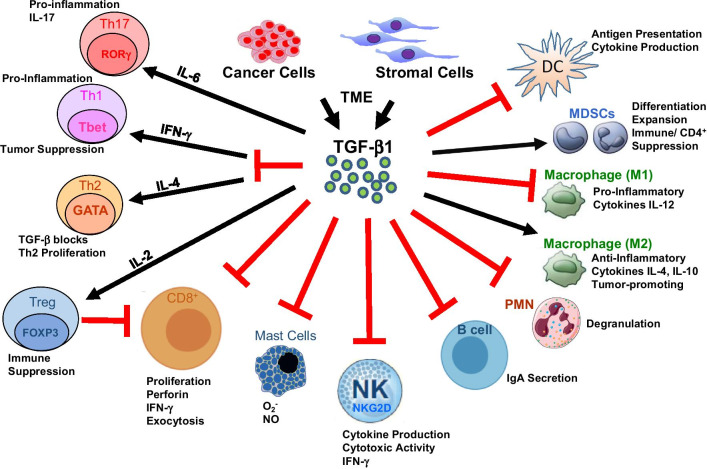
Effects of TGF-β on Antitumor Immunity. TGF-β is produced by multiple cell types within the TME. TGF-β can increase (black arrow) or decrease (red block) the proliferation and functional activity of immune effectors with tumor-promoting or tumor-suppressive outcomes. TGF-β enhances pro-inflammatory Th17 cells along with IL-6. TGF-β also blocks the IFN-γ-mediated induction of pro-inflammatory Th1 cells as well as the IL-4-dependent production of Th2 cells to decrease tumor suppression. TGF-β induces naïve T cell differentiation into Tregs and Treg expansion with IL-2. Tregs then reduce CD8^+^ T cell development and expansion to increase immune suppression. TGF-β also reduces the production of mast cells (to reduce superoxide and NO production), natural killer (NK) cells (reduced cytokine and IFN-γ production), B cells (reduced IgA secretion), polymorphonuclear cells (PMNs, reduced degranulation), M1 macrophages (reduced pro-inflammatory cytokines, e.g., IL-12) and reduced dendritic cells (DCs) leading to reduced antigen presentation and cytokine production. TGF-β promotes the differentiation and expansion of MDSCs as well as the production of M2 macrophages leading to increased anti-inflammatory cytokines IL-4 and IL-10

TGF-β modulates the level and functional activity of numerous immune cell types to exert systemic antitumor immune suppression and inhibit host immunosurveillance [17–19], (Fig. 2). In addition to direct effects on tumor cells, TGF-β regulates infiltration of inflammatory, immune cells and cancer-associated fibroblasts in the TME [20–25]. Neutralizing TGF-β enhances CD8^+^ T-cell- and NK-cell-mediated antitumor immune responses and increases neutrophil-attracting chemokines resulting in recruitment and activation of neutrophils with an antitumor phenotype. TGF-β also inhibits naïve T cell differentiation into the Th1 phenotype that is the most prominent and best-characterized T cell response against cancer. TGF-β further inhibits T cell proliferation and effector function by silencing expression of IL-2, the cytokine that elicits subsequent CD4^+^ T cell proliferation. TGF-β signaling directly inhibits the cytotoxic program of CD8^+^ T cells through mechanisms which stimulate SMADs and transcription factor ATF1 to repress granzyme B and IFN-γ, involved the lytic function of CD8^+^ T cells [25].

Regulatory T cells (Tregs) suppress the activity of effector T cells to maintain immune homeostasis, and their number is increased in the TME. Tregs facilitate immune evasion and are stimulated to expand and differentiate by TGF-β. TGF-β in combination with IL-2 enforces a suppressor phenotype in ex vivo naïve CD4^+^ T cells by triggering expression of FOXP3, the master transcription factor of the Treg program [26, 27]. Treg functionality is mediated by TGF-β secretion, and *Foxp3* upregulation by TGF-β is mediated by both Smad2 and Smad3 [28]. An environment rich in pro-inflammatory cytokines counteracts TGF-β-driven induction of Tregs as it favors differentiation of CD4^+^ T cells toward an effector phenotype [29–32].

TGF-β signaling suppresses the generation and function of NK cells by silencing IFN-γ and Th1 transcription factor T-bet expression in NK cells, thus inhibiting Th1 responses [33–37]. Pro-inflammatory signals counteract this mechanism by decreasing TGF-β II levels and suppressing downstream SMAD signaling in NK cells. TGF-β signaling inhibits the expression of NKG2D and NKp30, two surface receptors of NK cells that mediate the recognition of stressed and malignant transformed cells [36, 37]. Dendritic cells (DCs) are highly potent antigen-presenting cells and play a key role in tumor immunity and in the regulation of Th1 and Treg-mediated immune responses [38–42]. TGF-β inhibits the antigen presentation capability of DCs in vitro by suppressing MHCII gene expression. Cancer cells direct DCs to secrete TGF-β, which in turn induces conversion of naïve CD4^+^ T cells into Tregs.

The TME also polarizes macrophages toward a M2 phenotype with anti-inflammatory, immunosuppressive and pro-angiogenic functions [43–47]. Tumor-associated macrophages (TAMs) produce TGF-β and subsets of macrophages that can mobilize active TGF-β through the activity of integrin αv β8 and MMP1. TGF-β acts as chemoattractant for monocytes to the sites of inflammation and upregulates adhesion molecules that enable monocyte attachment to the ECM. Monocytes differentiate into perivascular macrophages and facilitate tumor cell extravasation by promoting blood vessel leakiness. A TGF-β-rich TME may contribute to immune evasion by dampening the inflammatory functions of macrophages.

TGF-β1-mediated programming of nascent myeloid-derived suppressor cells (MDSCs) leads to a potent antitumor phenotype potentially suitable for adoptive immunotherapy [48, 49]. TGF-β is involved in controlling MDSC differentiation and immunoregulatory function in vivo, and MDSCs regulate T cell immunity. TGF-β increases expansion of the monocytic MDSC (Mo-MDSC) population, expression of immunosuppressive molecules by MDSCs and the ability of MDSCs to suppress CD4^+^ T cell proliferation [50].

TGF-β is a pleiotropic cytokine with a crucial function in mediating immune suppression and evasion of immunosurveillance in the TME. TGF-β produced by T cells has been shown to be an important factor for suppressing antitumor immune responses, but the precise role of tumor-derived TGF-β has been poorly understood. Knockdown of tumor-derived TGF-β using shRNA resulted in dramatically reduced tumor size, slowed tumor formation, prolonged survival of tumor-bearing mice and inhibited metastatic growth [51]. Mechanistically, reducing the number of MDSCs and CD4^+^Foxp3^+^ Treg cells, enhanced IFN-γ production by CTLs. Knockdown of tumor-derived TGF-β also significantly reduced the conversion of naive CD4^+^ T cells into Treg cells in vitro. Knockdown of TGF-β also suppressed cell migration. TGF-β has also been found to be particularly important for the maintenance of low affinity CD4^+^ T cells [52]. In the absence of TGF-β, IL-7Rα expression positively correlated with TCR affinity, as TGF-βRII-deficient T cells bearing higher affinity TCRs expressed increased amounts of IL-7Rα and, thus, exhibited better homeostatic survival than their lower affinity counterparts.

Chimeric antigen receptor–modified T cell (CAR T cell) therapy has proven to be a promising approach against eliminating solid tumors, but the immunosuppressive TME remains a significant obstacle. Knocking out the endogenous TGF-β receptor II (*TGFBR2*) in CAR T cells with CRISPR/Cas9 technology was an approach used to reduce the induced Treg conversion and prevent CAR T exhaustion. *TGFBR2*-edited CAR T cells had better in vivo tumor elimination efficacy, both in cell line–derived xenograft and patient-derived xenograft solid tumor models, whether administered locally or systemically [53]. Knocking out endogenous *TGFBR2* greatly improved the in vivo function of CAR T cells in TGF-β–rich TME. The potency of CAR T cells directed to prostate-specific membrane antigen (PSMA) was also enhanced by co-expression of a dominant-negative TGF-βRII (dnTGF-βRII) [54]. Expression of the dnTGF-βRII in CAR T cells increased lymphocyte proliferation, enhanced cytokine secretion, promoted T cell resistance to exhaustion and increased tumor eradication in aggressive human prostate cancer models.

## TGF-β pathway biomarkers to predict cancer risk and therapeutic response

### Blood, serum and tissue markers

Measurement of TGF-β pathway components in blood, serum and tissue represents a rapid, accurate and inexpensive approach to determine cancer risk, stratify patients into treatment populations and predict therapeutic response. Early detection of tumors generally improves patient survival, and patients diagnosed at an advanced stage have poorer prognosis. Increased TGF-β levels within the primary tumor as well as high plasma levels of TGF-β correlate with poor prognosis in CRC patients [55, 56]. TGF-β expression is increased in pancreatic cancer and associated with poor prognosis [57, 58]. TGF-β is also highly expressed in HCC, and TGF-β1 levels are associated with disease progression and poor outcome [59]. While during the early phases TGF-β inhibits proliferation of premalignant hepatocytes, later it promotes stromal formation, EMT and invasion [60]. Elevated serum TGF concentration at diagnosis in MM patients may be a favorable predictor of response [61].

A nested case-controlled study using a large real-world population-based dataset exhibited that low serum TGF-β1 concentrations predict death from HCC, with a 160% increase in the risk per one SD decrease in the TGF-β1 concentrations [62], This association remained statistically significant even after adjusting for confounding factors and in a sensitivity analysis restricted to participants who survived for at least five years after the baseline survey, effectively eliminating the possibility that these patients had undiagnosed HCC at the baseline survey. The data demonstrate that low levels of TGF-β1 can identify patients who are at higher risk of developing HCC. Furthermore, the predictive value was significantly higher among HCV-positive patients. TGF-β1 may be a serum predictor that becomes altered well before the development of clinically detectable malignancy. The findings are in agreement with the general theory that TGF-β1 is frequently present in the tumor microenvironment and prevents premalignant progression during the beginning phases of carcinogenesis.

### Gene expression and transcriptomics

Signatures identified by gene expression and transcriptomic profiling may serve as diagnostic aids and improve predicting and treatment response compared to single biomarkers. The molecularly guided trials with specific treatment strategies in patients with advanced newly molecular defined subtypes of colorectal cancer (MoTriColor) consortium are performing three two-stage single-arm multi-center open-label phase II studies in molecular- and gene expression-selected mCRC populations. In one treatment cohort, patients with chemotherapy-resistant activated TGF-β signature-like tumors will have received 5FU or capecitabine in the first line of chemotherapy, usually combined with oxaliplatin. Newly identified biomarkers may be included to identify alternative combinations for patients susceptible of developing secondary resistance.

### Immune markers

Checkpoint inhibitor-based immunotherapies that target cytotoxic T lymphocyte antigen 4 (CTLA4) or the programmed cell death 1 (PD1) pathway have achieved impressive success in the treatment of different cancer types, but only a subset of patients derive clinical benefit [63]. TGF-β signaling pathway participated in cancer immune escape and ICI resistance. Concurrent TGF-β blockade might be a feasible strategy to enhance the efficacy of immunotherapy and relieve immune checkpoint inhibitor resistance [64]. Selective inhibition of TGF-β1 activation overcomes primary resistance to checkpoint blockade therapy by altering tumor immune landscape [65]. Biomarkers superior to tumor mutational burden would greatly enhance patient selection for immune checkpoint inhibitor therapies.

## Agents in clinical development to target the TGF-β pathway in oncology

Despite significant advancements in the diagnosis and treatment of certain cancer types, overall survival (OS) in many cancers has remained relatively constant. Poor initial response rates and the emergence of drug resistance remain significant challenges. Cancer investigators strive to develop more-effective treatments that have fewer side effects than do standard cancer therapies. A number of receptor kinase inhibitors, monoclonal antibodies (mAbs), ligand traps, antisense oligonucleotides and vaccines have been designed to target the TGF-β pathway and have been evaluated clinically (Fig. 1). Many of these of agents have been or are being evaluated in clinical trials to treat a number of different cancer types (Tables 1, 2).

**Table 1:** Completed clinical trials that evaluated TGF-β pathway antagonists in oncology

Study NCT Registry Number	Agent	Target(s)	Study Population	Number of Patients	Phase	Clinical Efficacy	Most Frequent Adverse Events
*Small molecule receptor kinase inhibitors*							
NCT02160106	Vactosertib	TGF-β RI	Solid tumors	29	I	Pharmacokinetics	
	(TEW-7197)			35	I	Dosing model	
NCT01682187	Galunisertib (LY2157299)	TGF-β RI	Advanced solid tumors	65	I	Glioma population ORR 14%	Thrombocytopenia thrombosis, dyspnea
NCT01582269	Galunisertib ± Lomustine	TGF-β RI	Refractory glioma	180	II	mOS: Galunisertib 8 m, Lomustine 7 m, Both 6.5 m	Fatigue, nausea, vomiting
NCT01220271	Temozolomide RT ± Galunisertib	CT/RT TGF-β RI	GBM	75	Ib/II	mOS 18.2 vs. 17.9 m	Fatigue, nausea constipation
NCT01373164	Galunisertib ± Gemcitabine	TGF-β RI Chemotherapy	Inoperable or metastatic pancreatic	170	I/II	mOS 8.9 vs. 7.1 m	Neutropenia, thrombocytopenia
NCT02154646	Galunisertib + Gemcitabine	TGF-β RI Chemotherapy	Inoperable or metastatic pancreatic cancer	9	I	ORR 0%	Elevated liver enzymes
NCT02734160	Galunisertib + Durvalumab	TGF-β RI PD-L1	Metastatic pancreatic cancer	32	I	ORR 3% mPFS 1.9	Elevated liver enzymes neutropenia
NCT02240433	Galunisertib + Sorafenib	TGF-β RI TKI, Angiogenesis	Metastatic HCC	14	I	ORR 9%	Hypophosphatemia Hand-foot syndrome
NCT01246986	Galunisertib	TGF-β RI	Metastatic HCC	147	II	mPFS: 2.7 m (part A) 4.2 m (part B)	Neutropenia
*Antibodies*							
NCT00356460	Fresolimumab	TGF-β1, β2, β3	Advanced melanoma Renal cell cancer	29	I	ORR 3 and 5% mPFS 2.75 m	Keratoacanthomas hyperkeratosis
NCT01472731	Fresolimumab RT	TGF-β1, β2, β3	Glioma	23	II	ORR 0%	Fatigue, anemia keratoacanthomas
NCT01646203	LY3022859	TGF-β RII	Advanced solid Tumors	14	I	Not reported	CRS
*Ligand Traps*							
NCT04296942	Bintrafusp alfa, BN-Brachyury, Entinostat, Adotrastuzumab Emtansine	TGF-β RII and PD-L1	Advanced Stage Breast cancer	19	I	ORR 21%	Bullous pemphigoid increased lipase, colitis
NCT03427411	Bintrafusp alfa	TGF-β RII and PD-L1	HPV-positive advanced solid tumors	120 (estimated enrollment)	II	ORR 39%	Colitis, hypokalemia gastroparesis
NCT02517398	Bintrafusp alfa	TGF-β RII and PD-L1	Pre-treated cervical tumors	25	I	ORR 28%	Hypokalemia
NCT02517398	Bintrafusp alfa	TGF-β RII and PD-L1	Refractory head and neck cancer	32	I	ORR 22%	Keratoacanthomas hyperglycemia
NCT02517398	Bintrafusp alfa	TGF-β RII and PD-L1	Pre-treated NSCLC	80	II	PD-L1 > 1%, ORR 40% PD-L1 > 80%,ORR 71%	Keratoacanthomas
NCT02517398	Bintrafusp alfa	TGF-β RII and PD-L1	Pre-treated esophageal adenocarcinoma	30	I	ORR 20%	Anemia, pain
NCT02517398	Bintrafusp alfa	TGF-β RII and PD-L1	Pre-treated gastric cancer	31	I	ORR 22%	Anemia, diarrhea, rash
NCT02517398	Bintrafusp alfa	TGF-β RII and PD-L1	Pre-treated biliary tract cancer	30	I	ORR 23%	ILD
NCT02517398	Bintrafusp alfa	TGF-β RII and PD-L1	Refractory colorectal cancer	29	I	ORR 3.4%	Anemia, fatigue enteritis
NCT02631070	Luspatercept	ActRIIb IgG1	Very low, low or intermediate risk MDS	229	III	Transfusion independent > 8 weeks asthenia, nausea, 38% vs. 13%	Fatigue, diarrhea,
*Antisense nucleotides*							
NCT00431561	Trabedersen vs. Temozolomide/ Lomustine	TGF-β2 mRNA	Recurrent/ Refractory glioma	142	IIb	6 m tumor control rate: Trabedersen 10uM: 33% Trabedersen 80uM: 20% Chemotherapy: 27%	Nervous disorders
NCT00844064	Trabedersen	TGF-β2 mRNA	Advanced tumors known to overproduce TGF-β2	62	I		
*Vaccines*							
NCT01058785	Belagenpumatucel-L	TGF-β2 Inhibition	NSCLC	75	II	ORR 15%	Pain, anemia fatigue
NCT00676507	Belagenpumatucel-L	TGF-β2 Inhibition	Inoperable or metastatic NSCLC	532	III	mOS 20 vs 17 m	Allergic reactions
NCT02574533	Vigil + Pembrolizumab	Vaccine Anti-PD-1	Advanced melanoma	2	I		

Summary of completed clinical trials utilizing TGF-β targeting agents in a variety of treatment refractory metastatic solid tumors including breast, esophageal, gastric, biliary, pancreatic, non-small cell lung cancer, melanoma, GBM and head and neck tumors. Nine trials utilized small molecule inhibitors, three utilized antibodies, ten utilized ligand traps, two utilized ASOs, and three utilized vaccination strategies

**Table 2:** Ongoing clinical trials to evaluate TGF-β pathway antagonists in oncology

Study NCT Registry Number	Agent	Target(s)	Study Population	Number of Patients	Phase	Clinical status
*Small molecule receptor kinase inhibitors*						
NCT03724851	Vactosertib + Pembrolizumab	TGF-β RI	Metastatic CRC, gastric, or GEJC adenocarcinoma	67	Ib/IIa	Active, recruiting
NCT03732274	Vactosertib + Durvalumab	TGF-β RI PD-L1	Advanced NSCLC	63	Ib/IIa	Active, recruiting
NCT04064190	Vactosertib + Durvalumab	TGF-β RI PD-L1	Urothelial cancer	48	II	Active, recruiting
NCT03143985	Vactosertib + Pomalidomide	TGF-β RI IMiD Agent (Cereblon)	Relapsed/ Refractory Multiple myeloma	18	Ib/IIa	Active, recruiting
NCT03698825	Vactosertib + Paclitaxel	TGF-β RI Tubulin/ Mitotic spindle	Second line therapy for metastatic gastric AC	62	Ib/IIa	Active, recruiting
NCT04103645	Vactosertib	TGF-β RI	Anemic, Ph-neg, MPN	37	II	Active, not recruiting
NCT04258072	Vactosertib naI-IRI/FL	TGF-β RI Chemotherapy	Metastatic pancreatic Adenocarcinoma	24	Ib/IIa	Active, not recruiting
NCT03802084	Vactosertib Imatinib	TGF-β RI BCR-abl	Advanced desmoid tumor	24	Ib/IIa	Active recruiting
NCT02452008	Galunisertib + Enzalutamide	TGF-β RI AR	Castration-resistant prostate cancer	60	II	Active, recruiting
NCT03206177	Galunisertib + Carboplatin/ Paclitaxel	TGF-β RI Chemotherapy	Ovarian carcinosarcoma	25	I	Active, recruiting
NCT02688712	Galunisertib + Chemo/RT	TGF-β RI	Locally advanced rectal cancer		II	Active, recruiting
NCT04031872	LY3200882 Capecitabine	TGF-β RI Chemotherapy	Advanced Chemotherapy Resistant Colorectal Cancer and an Activated TGF-beta Signature	31	I/II	Active, not recruiting
*Antibodies*						
NCT02581787	Fresolimumab + SBRT	TGF-β1, β2, β3 RT	Stage Ia/Ib NSCLC	60	I/II	Active, not recruiting
NCT03192345	SAR438459 + Cemiplimab	TGF-β1, β2, β3 PD-L1	Advanced solid tumors	350	I	Active, not recruiting
NCT04291079	SRK-181 + Anti-PD-L1	TGF-β1 PD-L1	Locally advanced or metastatic solid tumors	183	I	First-in-human, dose-escalation, dose expansion study to evaluate safety, tolerability, PK, PD
*Ligand Traps*						
NCT04349280	Bintrafusp alfa	TGF-β RII and PD-L1	Metastatic or Locally Advanced/Unresectable Urothelial Cancer With Disease Progression or Recurrence Following Treatment With a Platinum Agent	40	Ib	Active, recruiting
NCT04501094	Bintrafusp alfa	TGF-β RII and PD-L1	Checkpoint inhibitor naive urothelial carcinoma	75	II	Active, not yet recruiting
NCT04066491	Cisplatin/Gemcitabine +/- Bintrafusp alfa	Chemotherapy TGF-β RII and PD-L1	First-line Treatment of Biliary Tract Cancer	512	II/III	Active, recruiting
NCT04220775	SBRT Bintrafusp alfa	RT TGF-β RII and PD-L1	Recurrent or Second Primary Head and Neck Squamous Cell Cancer	21	I/II	Active, not yet recruiting
NCT03833661	Bintrafusp alfa	TGF-β RII and PD-L1	Locally Advanced or Metastatic Biliary Tract Cancer Who Fail or Are Intolerant to First-line Platinum-Based Chemotherapy	159	II	Active, not recruiting
NCT03524170	Bintrafusp alfa	TGF-β RII and PD-L1	Metastatic Hormone Receptor Positive, HER2 Negative Breast Cancer	20	I	Active, recruiting
NCT04296942	Bintrafusp alfa	TGF-β RII and PD-L1	Advanced Stage Breast Cancer (BrEAsT)	65	I	Active, recruiting
	Brachyury-TRICOM Ado-trastuzumab Emtansine	Vaccines HER2 HDAC deacetylase				
NCT03436563	Bintrafusp alfa	TGF-β RII and PD-L1	Metastatic colorectal cancer or advanced solid tumors with MSI-high	74	I/II	Active, recruiting
NCT03840915	Platinum regimen Bintrafusp alfa + and PD-L1	Chemotherapy TGF-β RII	Stage IV Non-small Cell Lung Cancer	70	Ib/II	Active, not recruiting
NCT03840902	Chemo-RT Bintrafusp alfa +	Chemo-RT TGF-β RII	Unresectable Stage III Non-small Cell Lung Cancer. Bintrafusp alfa with concurrent chemoradiation followed by bintrafusp vs. concurrent chemoradiation + placebo followed by durvalumab	350	III	Active, recruiting
*Vaccines*						
NCT02346747	Vigil	Vaccine	Stage IIIb, IIIc, IV high-grade papillary, serous, clear cell, serous ovarian cancer.	91	II	Active, not recruiting
NCT03073525	Vigil Atezolizumab	Vaccine PD-L1	Stage IIIb, IV ovarian cancer. Patients had tumor harvested at surgery and successful manufacturing of Vigil but were ineligible for CL-PTL-119 (the VITAL study) or previously randomized to placebo.	25	II	Active, not recruiting
NCT02725489	Vigil Durvalumab	Vaccine PD-L1	Advanced Women’s cancers. Confirmed diagnosis of women's cancer, inclusive, but not limited to breast, ovarian, fallopian tube, primary peritoneal, uterine, cervical, endometrial, that is locally advanced or metastatic for which the projected response rate to durvalumab is 15% or less.	13	II	Active, not recruiting
NCT02511132	Vigil	Vaccine	Metastatic Ewing's sarcoma refractory or intolerant to at least 1 prior line of systemic chemotherapy.	22	IIb	Active, not recruiting
	vs. Gem/docetaxel	Chemotherapy				(Part A)
	+ Temozolomide/ Irinotecan				II	(Part B)
NCT03842865	Vigil	Vaccine	Expanded Access Trial of Vigil (Bi-shRNAfurin and GMCSF Augmented Autologous Tumor Cell Immunotherapy) in Advanced Solid Tumors including Ewing's sarcoma, Ewing's tumor metastatic, Ewing's sarcoma metastatic, advanced gynecological cancers, ovarian, cervical, and uterine cancers.	40	Expanded Access	Temporarily not available

Summary of ongoing clinical trials that are utilizing TGF-β targeting agents in solid tumor as well as hematological malignancies including MM and MPNs. Twelve trials utilized small molecule inhibitors, three utilized antibodies, nine utilized ligand traps, and five utilized vaccination strategies

### Small molecule inhibitors of TGF-β receptor kinase activity

Several TGF-β R kinase inhibitors have been designed to bind the ATP-binding domain of TGF-β R kinase and inhibit ATP kinase activity and block the downstream signaling cascade [66, 67].

**Fig. 3:**
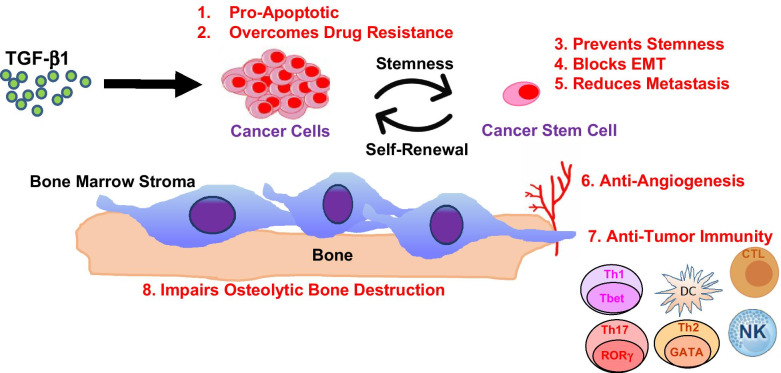
Tumor Intrinsic and Extrinsic Effects of Vactosertib. Vactosertib exerts potent antitumor effects (indicated in red) directly on cancer cells as well as on a number of other cell types

**Vactosertib** (TEW-7197, MedPacto) is an orally available inhibitor of the kinase activity of TGF-β RI/ALK-5 (IC_50_ = 12.9 nM) [68, 69]. Vactosertib also inhibits ALK-2 and ALK-4 with an IC_50_ value of 17.3 nM. In murine models, Vactosertib limits the growth and suppresses progression of many solid tumor types and as well as the plasma cell neoplasm MM. Mechanistically, Vactosertib acts pleiotropically on multiple cell types through tumor intrinsic and extrinsic mechanisms (Fig. 3). The safety and efficacy of Vactosertib against a number of different cancer types have been reported in a number of completed trials (Table 1).

A first-in-human phase I dose escalation study investigated the safety, tolerability and pharmacokinetics of Vactosertib in patients with advanced, refractory solid tumors (NCT02160106) [70, 71]. Vactosertib pharmacokinetics were dose proportional when administered once daily for five days. Based upon a relatively short half-life, it was postulated that Vactosertib should be administered twice or thrice daily to maintain a concentration above a minimum effective level over dosing interval. A two-compartment linear model with first-order absorption and absorption lag time adequately described the population pharmacokinetics [70].

An open-label, multi-center study assessed the safety, tolerability, pharmacokinetics and antitumor activity of Vactosertib in combination with Pembrolizumab (Keytruda, Merck) in patients with either metastatic colorectal or gastric cancer or gastroesophageal junction adenocarcinoma (NCT03724851, [72]). Study results indicated objective response rates (ORRs) of 16.7% and 33.3%, respectively, for colon cancer patients who failed entire body anticancer treatment based on RECIST and iRECIST (primary and secondary anticancer evaluation indices). Colon cancer patients who participated in clinical tests demonstrated a microsatellite stable genotype, which previously had a non-existent objective response rate with pembrolizumab alone. A decrease in the colon cancer tumor marker CEA value was observed in half of the patients that received Vactosertib and suggested that tumor was effectively reduced in patients as treatment continued.

Combined inhibition of immune checkpoint and TGF-β signaling is a promising therapeutic strategy since these key pathways have independent but complementary immunosuppressive functions. Safety and efficacy of Vactosertib combined with Durvalumab (Imfinzi, Medimmune, AstraZeneca), a human mAb directed against programmed death-ligand 1 (PD-L1), was evaluated in patients with metastatic non-small cell lung cancer (NSCLC) and urothelial carcinoma (NCT03732274, [73]). The trial was conducted on 15 NSCLC patients and a 16.7% ORR was observed, even though patients demonstrated < 25% of PD-L1 expression in tumors. Importantly, imfinzi monotherapy demonstrated an ORR of 2.8% clinical test conducted on the same patient group. A phase II, open-level, non-randomized single-arm study will determine whether the administration of Vactosertib with Durvalumab increased the overall response rate in patients with urothelial cancers that fail to achieve a CR with anti-PD-1/PD-L1-based regimens (NCT04064190). The combination of Vactosertib plus Durvalumab is well-tolerated and without safety concerns.

A phase II study will determine the recommended dose and evaluate the safety of Vactosertib in combination with nanoliposomal irinotecan (NaI-IRI) with 5-FU and leucovorin (FL) in patients with metastatic pancreatic ductal adenocarcinoma (PDAC) who have failed first-line gemcitabine (GEM) and nab-paclitaxel (NCT04258072). A phase I/II, open-label, non-randomized, multicenter study will evaluate the clinical activity of Vactosertib plus Imatinib in desmoid tumors (NCT03802084). Based upon impressive preclinical and translational studies, Vactosertib has been combined with the immunomodulatory agent Pomalidomide (Pom, Pomalyst, Bristol-Myers Squibb) in a phase Ib/IIa study to treat participants with relapsed and/or refractory MM (NCT03143985) [74, 75]. Results indicated that the combination was safe and that progression-free survival (PFS) was 80%, higher than historical controls seen with Pom alone (PFS = 20%) or that seen with Pom and corticosteroids (PFS = 40%).

A dose escalation, proof-of-concept study of Vactosertib monotherapy in patients with myelodysplastic syndromes (MDS) has also been reported (NCT03074006). A prospective, open-label, multicenter, phase I/II study of Vactosertib in patients with low and intermediate risk of MDS evaluated hematological improvement, maximum tolerated dose, bone marrow response and quality of life. Preclinical and clinical results indicated that TGF-β1 levels were increased in subset of MDS samples and Vactosertib treatment led to reversal of hematopoietic alteration upon exposure to MDS serum in vitro and increased blood counts in vivo [76, 77].

A two-tiered, single-arm, phase II trial of Vactosertib for anemic myeloproliferative neoplasm (MPN) patients was recently designed (NCT04103645) and will assess how well Vactosertib works in MPN patients to improve anemia and provide new information about how this agent blocks TGF-β signaling in MPN cells. In turn, the study will determine whether Vactosertib can block MPN cells and their effects on bone marrow, e.g., fibrosis.

An open-labeled, multicenter phase Ib/IIa, single-arm study was designed to evaluate the safety and tolerability of Vactosertib in combination with weekly paclitaxel as second-line treatment for metastatic gastric adenocarcinoma patients (NCT03698825). A report is that paclitaxel (Taxol; Bristol-Myers Squibb Co.) significantly suppressed the TGF-β /Smad signaling pathway by inhibiting Smad2 phosphorylation in the peritoneum [78]. A multi-center, open-label, phase Ib clinical was designed to evaluate the safety, tolerability and exploratory efficacy of Vactosertib in combination with FOLFOX in patients with PDAC who failed first-line GEM and nab-Paclitaxel (NCT03666832).

**Galunisertib** (LY21557299, Eli Lilly & Co.) is a TGF-βRI kinase inhibitor   that has been shown to reduce the growth of lung and breast cell lines and was safe in patients with various solid tumors in phase I studies [79–81]. In a phase II study of galunisertib in combination with lomustine, compared to lomustine with placebo, in patients with recurrent glioma failed to demonstrate improved OS relative to placebo plus lomustine (NCT01582269). In a separate phase II study, 40 patients with hepatocellular carcinoma (HCC) that had progressed on or were ineligible to receive sorafenib (Bayer, Onyx) were treated with intermittent dosing of galunisertib (14 days on/14 days off) [82]. HCC patients with normal α-fetoprotein and with TGF-β1 reduction improved in OS compared to patients with non-TGF-β1 reduction (NCT01246986). In a phase II study of patients with unresectable pancreatic cancer, patients treated with galunisertib in combination with GEM improved OS (10.9 vs. 7.2 months) compared to those treated with GEM and placebo (NCT01373164).

The incentive to move galunisertib forward was based largely on the development of an intermittent dosing schedule by predictive pharmacology, pharmacodynamic markers and preclinical toxicology models [83]. The optimal effective dosing schedule that can induce antitumor activity, but lacks cardiac toxicity, consists of iterative cycles (28 days/cycle) comprised of two-week treatment with galunisertib followed by two weeks without drug. The protocol was applied to patients with glioma, and serious cardiac toxicity was not observed [84]. In metastatic PDAC, targeting TGF-β signaling could be a rational approach since it is involved in tumor progression and has been associated with poor prognosis [85]. A randomized phase II study assigned 156 patients to receive galunisertib plus GEM or placebo plus GEM in stage II-IV unresectable PDAC. The combination of galunisertib and GEM resulted in improved OS and PFS and a manageable toxicity profile compared to that of placebo and GEM [85, 86]. 

**LY3200882** (Eli Lilly) is a next-generation, potent and highly selective ATP competitive inhibitor of the TGF-βRI serine-threonine kinase domain [87]. In a multicenter, non-randomized, open-label study, dose-escalation study of LY3200882 monotherapy, the recommended phase II dose was established at 50 mg bid at two weeks on/two weeks off for patients with advanced or metastatic cancers were treated with (NCT02937272) [88]. Five cohorts of patients with advanced or metastatic cancer following progression on standard therapy received LY3200882 twice daily at increasing doses. Tumor types included were glioma (*n* = 15), GBM (*n* = 5), pancreatic (*n* = 3), cervical (*n* = 3), chondrosarcoma (*n* = 2), appendiceal (*n* = 1) and colorectal adenocarcinoma (*n* = 1). Findings showed that a twice-daily dose of 50 mg at two week-on/two week-off schedule was optimal. Moreover, dose-limiting toxicities were not observed, and all treatment-related adverse events were of grade 1 or 2. Across all cohorts, there was one partial response in the 50 mg cohort—six cases of stable disease, 11 with progressive disease and 12 with non-evaluable disease. The overall response rate (ORR) was 3.3% (*n* = 1), and disease-control rate was 23.3% (*n* = 7). Additionally, an 85.7% tumor reduction, assessed by response assessment in neuro-oncology criteria, was reported in one patient with EGFR-mutant, CDK4-amplified, IDH1/2 wild-type and MGMT-methylated GBM. The patient was treated at 50 mg bid and has remained on treatment for more than 11 months.

**LY573636** (Tasisulam, Eli Lilly) LY573636 is a TGF-β RI inhibitor that exhibited broad spectrum of preclinical antitumor activity [89]. LY573636 was evaluated in a phase I dose-escalation study in patients with advanced or refractory solid tumors. The primary objective was to determine the recommended phase II dose of tasisulam; secondary objectives were to characterize the toxicity and pharmacokinetic profiles of tasisulam and to discern whether there was any antitumor activity [89]. Twenty-six patients were enrolled. No dose-limiting toxicities (DLTs) were observed until cohort 3 (grade 3 hyperbilirubinemia). Interim PK analyses of this and another ongoing phase I study suggested that a lower dose after cycle 1 was necessary for doses ≥ 2,500 mg because of the long half-life of tasisulam (~ 14 days). When administered as a flat-dose, 24-h infusion, the MTD of drug was a loading dose of 2,500 mg followed by a chronic dose of 1,750 mg, every 28 days. Consistent with the profile of the two-hour infusion in clinical development, bone marrow suppression was the major DLT.

**A83-01** is a small molecule TGF-β RI/ALK-5 kinase inhibitor with an IC_50_ of 12 nM [90]. Aberrant hepatic growth factor (HGF)/c-MET upregulation and activation is frequently observed in bladder cancer correlating with cancer progression and invasion. However, the mechanisms underlying HGF/c-MET-mediated invasion in bladder cancer remain poorly defined. As part of a negative feedback loop, SMAD7 binds SMURF2 targeting the TGF-β receptor for degradation. SMAD7 then acts as a SMURF2 agonist by disrupting interactions within SMURF2. Recently, Sim et al. showed that HGF stimulates TGF-β signaling through c-SRC-mediated phosphorylation of SMURF2 resulting in loss of SMAD7 binding and enhanced SMURF2 C2-HECT interaction that inhibits SMURF2 and enhances TGF-βR stabilization [91].

**LY2109761 **(Eli Lilly) is an orally active, selective TGF-βRI/II inhibitor with Ki values of 38 nM and 300 nM, respectively [92]. It is metabolically stable and suitable for in vivo studies. The efficacy of LY2109761 on tumor growth, survival and reduction of metastasis was evaluated in an orthotopic murine model of pancreatic cancer. LY2109761, in combination with GEM, significantly reduced tumor burden, prolonged survival and reduced spontaneous abdominal metastases [93].

**LY364937** (Eli Lilly), ** Ki26894** (Kirin Brewery Company). Treatment with the TGF-βRI/ALK-5 inhibitor LY364937 led to a reduction in formation of early bone and lung metastases in a MDA-MB-435-F-L orthotopic xenograft model into nude mice [94]. Ki26894 is another TGF-β type I receptor kinase inhibitor that has shown efficacy against breast and gastric cell lines and xenografts in a mouse cancer model [95, 96].

**LY580276** (Eli Lilly) has been shown to block EMT and tumor cell migration in pancreatic cancer and mouse mammary epithelial cells, respectively [97, 98].

**SB-431542 and SB-505124** (GlaxoSmithKline) are noteworthy TGF-β R kinase inhibitors in pre-clinical development. These TGF-β RI inhibitors block Smad2/3 phosphorylation. The expression of c-myc, which is downregulated by TGF-β in many cell types, was upregulated in MG63 human osteosarcoma cells, suggesting that upregulation of c-myc expression meay be the mechanism of blocking growth suppressing signals in of TGF-β in MG63 cells [99]. Inhibitors for a TGF-β type I receptor kinase, SB-431542 and Ki26894, potently enhanced osteoblast differentiation from bone marrow stromal cells as well as MC3T3-E1 cells. The TGF-β inhibition was able to restore osteoblast differentiation suppressed by MM cell conditioned medium as well as bone marrow plasma from MM patients  [100]. However, these inhibitors are not specific, are relatively unstable and generate unpredictable outcomes with unwanted, adverse side effects.

**SD-093 and SD-208** are also in pre-clinical development and exhibit antimetastatic effects against gliomas and melanomas [101, 102] and blocked EMT and tumor cell migration in pancreatic cancer [103].

**IN-1130** (In2Gen) that also inhibits TGF-βRI, ALK-4 and ALK-7 signaling decreased tumor growth of prostate cancer xenografts in mice and [104] and inhibited lung metastasis of breast cancer and increased overall survival (OS) of the mice [105].

### TGF-β-directed antibodies to block ligand activation or prevent ligand receptor binding

Antibodies are used to interfere with TGF-β ligand binding to its cognate receptor as well as to block activation of latent TGF-β. Both steps are critical for TGF-β to elicit its pro-tumorigenic and immune suppressive responses.

**SRK181-mIgG1** (Scholar Rock). Cancer immunotherapy, including immune checkpoint blockade, has achieved increased prominence in the recent years [65]. Despite breakthroughs achieved with cancer checkpoint therapy, only a fraction of patients respond to anti-PD-1 therapy because of primary or secondary (acquired) resistance. Studies have suggested that inhibition of TGF-β may help overcome resistance to immune checkpoint blockade (NCT04291079). Conventional TGF-β antagonists do not discriminate among the three isoforms of TGF-β. It was reasoned that a lack of isoform selectivity may underlie the toxicities observed in both preclinical and human studies. To achieve improved safety and a therapeutic window enabling higher dosing, antibodies were designed and aimed at selectively targeting the latent TGF-β1 complex, without meaningfully affecting other isoforms. Retrospective analyses of clinical tumor samples identified TGF-β1 as the most prevalent TGF-β isoform in solid cancers. Preclinical results demonstrated that the highly selective inhibition of TGF-β1 activation with SRK 181-mIgG1 overcame primary resistance to checkpoint inhibitors. SRK181-mIgG1 is a potent and highly selective inhibitor of the TGF-β1 isoform and is an investigational product candidate being developed to overcome primary resistance to checkpoint inhibitor therapy, such as anti-PD-(L)1 Abs. Results demonstrated the effectiveness and safety of the agent with immune checkpoint blockade in mouse cancer models.

**Fresolimumab** (GC1008, Genzyme) is a human mAb that neutralizes TGF-β1 and TGF-β2 that was tested in a phase I trial of 28 patients with malignant melanoma and one patient with RCC. Seven patients showed partial response, four developed reversible cutaneous keratoacanthomas and SCCs, and one patient acquired hyperkeratosis as unwanted side effects [106].

**LY3022859** (Eli Lilly), an anti-TGF-βRII mAb, inhibits receptor-mediated TGF-β signaling activation and was tested in a phase I trial composed of 14 patients with advanced solid tumors. The maximum dose tolerance was not determined. Dose escalation beyond 25 mg/dose was considered unsafe due to negative symptoms, e.g., uncontrolled cytokine release, despite prophylaxis. Unfortunately, a safe dose at which the drug was active was not determined [107].

**264RAD** (AstraZeneca) is a human mAb that targets αvβ6 integrin that has a key role in activation of latent TGF-β, has been shown to reduce tumor growth and metastasis in vivo [108, 109]. Targeting αvβ6 with 264RAD antibody alone or combined with trastuzumab (Herceptin, Genentech), a human epidermal growth factor receptor 2 (EGFR) neutralizing Ab, can benefit patients with high-risk, trastuzumab-resistant disease [110].

**1D11** (Genzyme Corp., Sanofi) binds TGF-β1, 2 and 3, which suppresses lung metastasis in a breast cancer mouse model, by increasing in the antitumor response of CD8^+^ T cells [111].

**2G7** (Genentech) is a second mAb that has shown efficacy in preclinical trials to inhibit breast cancer metastasis by increasing NK cell activity [112, 113].

### TGF-β ligand traps

**AVID200** (BMS) is an engineered TGF-β ligand trap comprised of TGF-βR ectodomains fused to a human Fc domain. AVID200 has antibody-like properties and is 1,000 times more selective for neutralizing TGF-β1 and TGF-β3, compared to TGF-β2 [114]. AVID200 does not target TGF-β2, which is a positive regulator of hematopoiesis and normal cardiac function, and blockade of TGF-β2 is undesirable. Since TGF-β1 and TGF-β3 are negative regulators of hematopoiesis, while TGF-β2 is a positive regulator of hematopoiesis, the unique isoform selectivity profile of AVID200 makes it an attractive agent for the treatment of MDS-associated anemia. The ability of AVID200 to selectively target TGF-β1 and TGF-β3 positions it to be an effective and well-tolerated therapeutic in oncology.

**Bintrafusp alfa** (GSK-4045154, M7824, MSB0011359C, EMD Serono/GlaxoSmithKline/ Merck KGaA) is an innovative first-in-class bifunctional fusion protein composed of a mAb against PD-L1 fused to the extracellular domain of the TGF-β receptor II [115]. In the 3 + 3 dose-escalation component of phase I study (NCT02517398), eligible patients with advanced solid tumors received Bintrafusp alfa once every 2 weeks until confirmed progression, unacceptable toxicity or trial withdrawal. Bintrafusp alfa demonstrated manageable safety profile in patients with heavily pre-treated advanced solid tumors. A phase 1, open-label trial included 80 patients with advanced NSCLC that progressed after platinum doublet therapy or platinum-based adjuvant or neoadjuvant treatment and those who also had not received previous immunotherapy. Patients were randomized at a one-to-one ratio to receive Bintrafusp alfa 500 mg or the recommended phase 2 dosage of 1200 mg every 2 weeks [116]. Bintrafusp alfa had encouraging efficacy and manageable tolerability in platinum-treated NSCLC patients. While pembrolizumab received accelerated FDA approval based upon ORR and duration of response (DOR) for patients with recurrent/metastatic cervical cancer with tumors that express PD-L1, the ORR of 14.3% suggests that there remains a significant unmet need for patients with previously treated advanced cervical cancer unselected for PD-L1. A multi-center, global single-arm phase II study (NCT04246489) will assess the clinical activity and safety profile of Bintrafusp alfa in platinum-experienced cervical cancer [117].

**Luspatercept** (Luspatercept-aamt, Acceleron) is a first-in-class erythroid maturation agent being developed to treat patients who have serious blood disorders associated with ineffective erythropoiesis. It is a recombinant fusion protein derived from human activin receptor type IIb (ActRIIb) linked to a protein derived from IgG. Luspatercept binds TGF-β ligands to reduce SMAD2 and SMAD3 signaling. Luspatercept was shown to reduce the severity of anemia in patients with lower-risk MDS with ring sideroblasts who had been receiving regular red-cell transfusions and who had disease that was refractory to erythropoiesis-stimulating agents or who had discontinued such agents [118].

**Soluble TGFβRII (sTGF-βRII)-Fc and soluble betaglycan (sBetaglycan)-Fc** were constructed as Fc fusion proteins, in which immunoglobulin fragment crystallizable (Fc) was fused to the extracellular domain of TGF-βRII and betaglycan, respectively [119]. In a manner similar to that of neutralizing antibodies, targeting TGF-β with sTGF-βRII-Fc and sBetaglycan-Fc also reduced tumor growth and metastasis in preclinical models. TβRII-Fc was shown to induce apoptosis in primary tumors and reduced tumor cell motility, intravasation and lung metastases from transplanted 4T1 and EMT-6 mammary tumors in syngeneic mice [120].

### ASOs targeting the TGF-β pathway

Antisense oligonucleotides (ASOs) offer a novel approach to specifically target and inactivate genes involved in cancer progression, especially those that are difficult to inhibit by small molecules or mAbs [121]. Due to their instability, nanoparticles and chemically modified ASOs have been developed to improve delivery and stability of oligonucleotides to target cells or tissues [122, 123].

**AP12009** (Trabedersen, Antisense Pharma GmbH/Isarna) targets TGF-β2 expression and is being studied to treat malignant glioma, pancreatic carcinoma and malignant melanoma with an immunotherapy approach. After providing preclinical proof-of-concept results, the safety and efficacy of AP12009 were assessed in an open-label phase I/II dose-escalation study for recurrent and/or refractory high-grade glioma patients [124, 125]. Median survival time after recurrence exceeded the current literature data for chemotherapy. AP12009 has undergone or is currently use in phase III trials against astrocytoma (NCT00761280).

**AP11014 and AP15012** are antisense molecules used in pre-clinical trials for treatment of non-small cell lung cancer, prostate carcinoma, CRC and MM, respectively [56, 126].

### Vaccine-based approaches to modulate TGF-β signaling

**Vigil **(Vital, Gradalis) is composed of autologous tumor cells harvested from the patient at the time of initial de-bulking surgery which are then transfected extracorporeally, with a plasmid encoding the gene for the immune-stimulatory cytokine GM-CSF, and a bifunctional short hairpin RNA (shRNA) which specifically knocks down expression of furin, a critical convertase responsible for production of TGF-β1 and TGF-β2. GM-CSF enhances surface antigen expression, making cancer cells more visible to the patient’s immune system and further stimulates the immune system by recruiting and maturing antigen-presenting cells, e.g., DCs. Vigil (Vital) is being investigated as a maintenance therapy in patients with high-risk stage 3b-4 ovarian cancer in a multicenter, randomized, double-blind, placebo-controlled, phase II/III trial (NCT02346747). The study involves more than 80 patients diagnosed with stage 3B, 3C or 4 high-grade papillary serous/clear cell/endometrioid ovarian, fallopian tube or primary peritoneal cancer. Patients were randomized to receive either vigil or placebo, and the primary goal is to determine patients’ recurrence-free survival (RFS) and overall survival (OS). Phase II of Vital in 42 cancer patients that demonstrated that Vigil is safe, leads to T-cell activation and improves RFS [127].

Another phase II crossover study (NCT03073525) is evaluating the safety and antitumor activity of vigil in women diagnosed with advanced gynecological cancers, e.g., ovarian, cervical and uterine. Vigil’s safety and antitumor activity, in combination with the immune checkpoint inhibitor atezolizumab (Tecentriq, Genentech), are being evaluated. Biomarkers are being used to measure antitumor systemic immune response generated either by Vigil alone, atezolizumab alone or the combination of the agents.

A pilot study (NCT02725489) is evaluating the safety, tolerability and efficacy of the combination of Vigil immunotherapy, and durvalumab (Imfinzi), a PD-L1 inhibitor, in women diagnosed with different types of advanced breast, ovarian, cervical, uterine, fallopian, primary peritoneal and endometrial cancer is recruiting patients. The main purpose of the study is to investigate the effects the combination treatment may have on patients, and whether or not the combination helps in slowing down the progression of the disease. An open-label pilot study (NCT02574533) examined the combination of vigil and pembrolizumab in inducing cancer-specific T-cell immunity in patients with incurable locally advanced or metastatic melanoma. Pembrolizumab is a humanized mAb which activates T-cell-mediated immune responses against tumor cells. The safety of vigil immunotherapy in combination with other drugs is also being evaluated in phase IIb trial (NCT02511132) involving patients with metastatic Ewing’s sarcoma that could not be treated with at least one prior line of systemic chemotherapy. Finally, a multicenter, expanded access protocol of intradermal autologous vigil in solid tumor patients is ongoing (NCT03842865).

**Belagenpumatucel-L** (Lucanix, NovaRx Corp.) was prepared by transfecting allogeneic non-small cell lung cancer (NSCLC) cells with a plasmid containing a TGF-β2 antisense transgene [128]. Patients that received belagenpumatucel-L had a dose-related survival advantage (NCT00676507) [129]. Tumor cells promote immunosuppressive Treg cell proliferation directly through TGF-β production or by DC conversion into regulatory cells that secrete TGF-β. TGF-β2 antagonizes natural killer (NK) cells, lymphokine-activated killer cells, and DC function and belagenpumatucel-L reduced TGF-β2-mediated immunosuppression [130–134].

### Shortcomings of TGF-β antagonists

The dual role of TGF-β in cancer highlights the need to better understand the contextual effects of this cytokine to better guide patient selection for the use of anti-TGF-β therapies in oncology. Furthermore, TGF-β is secreted by nearly every cell type, is therefore ubiquitous and regulates numerous critical physiologic processes in healthy cells. Limited mechanistic understanding of the dual, opposing actions of TGF-β as a tumor suppressor and tumor promoter remains a challenge in the development of TGF-β antagonists as cancer therapy. Combined with the pleiotropic activities of TGF-β and the lack of biomarkers, patient selection criteria and optimal dosing regimens are yet to be determined. However, many of these agents have limited activity when given as monotherapy, effective combinations with other anticancer strategies, e.g., immunotherapy, checkpoint inhibitors. Moreover, molecular biomarkers to stratify patient selection and treatment decisions are only beginning to emerge. Future clinical trials that incorporate bioinformatic tools and define biomarkers that identify patients who would benefit from TGF-β therapy are needed to advance the incorporation of TGF-β receptor antagonists into frontline cancer treatment.

## Conclusions

A number of TGF-β pathway antagonists have advanced to clinical trials and demonstrated acceptable safety profiles and significant therapeutic efficacy in cancer patients. Vactosertib, in combination with chemotherapy and immunotherapeutics, has been well tolerated and shown promising activity against a number of cancer types. AVID200 was safe, well tolerated with peripheral target engagement across the entire dosing period and led to TGF-β target modulation and immune activation. Bintrafusp alfa, the first-in-class bifunctional fusion protein TGF-β trap fused to a human immunoglobulin G1 antibody blocking PD-L1, had encouraging efficacy and manageable tolerability in patients with NSCLC previously treated with platinum. We conclude that TGF-β pathway antagonists, in combination with other modalities, are highly relevant since they provide a rapidly acting, cost-effective and feasible approach to improve cancer treatment. In addition, TGF-β pathway antagonists serve as a reasonable option for patients resistant to conventional therapies. While challenges remain, perspectives are enhanced by recently completed and ongoing clinical trials.

## Data Availability

Not applicable.

## Abbreviations

ALK: activin receptor-like kinase
ASO: antisense oligonucleotides
BMP: bone morphogenic protein
CDK: cyclin-dependent kinase
CSC: cancer stem cells
CTLA-4: cytotoxic T lymphocyte antigen-4
DC: dendritic cell
DLT: dose-limiting toxicity
EMT: epithelial to mesenchymal transition
EGFR: epithelial growth factor receptor
FC: fragment crystallizable
GBM: glioblastoma multiforme
GM-CSF: granulocyte macrophage colony-stimulating factor
GIC: glioma-initiating cells
NSCLC: non-small cell lung cancer
ORR: overall response rate
MDSC: myeloid-derived suppressor cells.
MPN: myeloproliferative neoplasm
MM: multiple myeloma
MDS: myelodysplastic syndrome
NK: natural killer
OS: overall survival
PDAC: pancreatic ductal adenocarcinoma
PFS: progression-free survival
PD-L1: programmed death ligand 1
RECIST: Response Evaluation Criteria in Solid Tumors
RFS: recurrence-free survival
Smad: * Caenorhabditis elegans Sma genes and the Drosophila mothers against decapentaplegic proteins*
TGF-β: transforming growth factor beta
Th: T helper
Treg: regulatory T cell
TKI: tyrosine kinase inhibitor
TME: tumor microenvironment

## References

[1] Moses HL, Roberts AB, Derynck R (2016). The discovery and early days of TGF-beta: a historical perspective. Cold Spring Harb Perspect Biol..

[2] Massague J (2008). TGFbeta in cancer. Cell.

[3] Moustakas A, Heldin CH (2009). The regulation of TGF beta signal transduction. Development.

[4] Massague J (2012). TGF beta signalling in context. Nat Rev Mol Cell Biol.

[5] Batlle E, Massague J (2019). Transforming growth factor-beta signaling in immunity and cancer. Immunity.

[6] Heldin CH, Moustakas A (2016). Signaling receptors for TGF-beta family members. Csh Perspect Biol..

[7] Tzavlaki K, Moustakas A (2020). TGF-beta signaling. Biomolecules.

[8] Smith AL, Robin TP, Ford HL (2012). Molecular pathways: targeting the TGF-beta pathway for cancer therapy. Clin Cancer Res.

[9] Blobe GC, Schiemann WP, Lodish HF (2000). Role of transforming growth factor beta in human disease. N Engl J Med.

[10] Bhowmick NA, Chytil A, Plieth D, Gorska AE, Dumont N, Shappell S, Washington MK, Neilson EG, Moses HL (2004). TGF-beta signaling in fibroblasts modulates the oncogenic potential of adjacent epithelia. Science.

[11] Ikushima H, Miyazono K (2010). TGFβ signaling: a complex web in cancer progression. Nat Rev Cancer.

[12] Tang J, Gifford CC, Samarakoon R, Higgins PJ (2018). Deregulation of negative controls on TGF-β1 signaling in tumor progression. Cancers (Basel).

[13] Boulay J-L, Mild G, Lowy A, Reuter J, Lagrange M, Terracciano L, Laffer U, Herrmann R, Rochlitz C (2003). SMAD7 is a prognostic marker in patients with colorectal cancer. Int J Cancer.

[14] Tang J, Gifford CC, Samarakoon R, Higgins PJ (2018). Deregulation of negative controls on TGF-β1 signaling in tumor progression. Cancers.

[15] Lindley LE, Briegel KJ (2010). Molecular characterization of TGF beta-induced epithelial-mesenchymal transition in normal finite lifespan human mammary epithelial cells. Biochem Bioph Res Commun.

[16] Giampieri S, Pinner S, Sahai E (2010). Intravital imaging illuminates transforming growth factor beta signaling switches during metastasis. Can Res.

[17] Yang L, Pang YL, Moses HL (2010). TGF-beta and immune cells: an important regulatory axis in the tumor microenvironment and progression. Trends Immunol.

[18] Dahmani A, Delisle JS (2018). TGF-beta in T cell biology: implications for cancer immunotherapy. Cancers..

[19] Gorelik L, Flavell RA (2002). Transforming growth factor-beta in T-cell biology. Nat Rev Immunol.

[20] Massague J (2000). How cells read TGF-beta signals. Nat Rev Mol Cell Biol.

[21] Sad S, Mosmann TR (1994). Single Il-2-secreting precursor Cd4 T-cell can develop into either Th1 or Th2 cytokine secretion phenotype. J Immunol.

[22] Gorelik L, Flavell RA (2000). Abrogation of TGF-β signaling in T cells leads to spontaneous T cell differentiation and autoimmune disease. Immunity.

[23] Wolfraim LA, Walz TM, James Z, Fernandez T, Letterio JJ (2004). p21(Cip1) and p27(Kip1) act in synergy to alter the sensitivity of naive T cells to TGF-beta-mediated G(1) arrest through modulation of IL-2 responsiveness. J Immunol.

[24] Sledzinska A, Hemmers S, Mair F, Gorka O, Ruland J, Fairbairn L, Nissler A, Muller W, Waisman A, Becher B (2013). TGF-beta signalling is required for CD4(+) T cell homeostasis but dispensable for regulatory T cell function. Plos Biol..

[25] Thomas DA, Massague J (2005). TGF-beta directly targets cytotoxic T cell functions during tumor evasion of immune surveillance. Cancer Cell.

[26] Donkor MK, Sarkar A, Savage PA, Franklin RA, Johnson LK, Jungbluth AA, Allison JP, Li MO (2011). T Cell surveillance of oncogene-induced prostate cancer is impeded by T cell-derived TGF-beta 1 cytokine. Immunity.

[27] Ahmadzadeh M, Rosenberg SA (2005). TGF-beta 1 attenuates the acquisition and expression of effector function by tumor antigen-specific human memory CD8 T cells. J Immunol.

[28] Yoon JH, Jung SM, Park SH, Kato M, Yamashita T, Lee IK, Sudo K, Nakae S, Han JS, Kim OH (2013). Activin receptor-like kinase5 inhibition suppresses mouse melanoma by ubiquitin degradation of Smad4, thereby derepressing eomesodermin in cytotoxic T lymphocytes. EMBO Mol Med.

[29] Chen WJ, Jin WW, Hardegen N, Lei KJ, Li L, Marinos N, McGrady G, Wahl SM (2003). Conversion of peripheral CD4(+)CD25(-) naive T cells to CD4(+)CD25(+) regulatory T cells by TGF-beta induction of transcription factor Foxp3. J Exp Med.

[30] Fantini MC, Becker C, Monteleone G, Pallone F, Galle PR, Neurath MF (2004). Cutting edge: TGF-beta induces a regulatory phenotype in CD4(+)CD25(-) T cells through Foxp3 induction and down-regulation of Smad7. J Immunol.

[31] Chen ML, Pittet MJ, Gorelik L, Flavell RA, Weissleder R, von Boehmer H, Khazaie K (2005). Regulatory T cells suppress tumor-specific CD8 T cell cytotoxicity through TGF-beta signals in vivo. Proc Natl Acad Sci USA.

[32] Takimoto T, Wakabayashi Y, Sekiya T, Inoue N, Morita R, Ichiyama K, Takahashi R, Asakawa M, Muto G, Mori T (2010). Smad2 and Smad3 are redundantly essential for the TGF-beta-mediated regulation of regulatory T plasticity and Th1 development. J Immunol.

[33] Laouar Y, Sutterwala FS, Gorelik L, Flavell RA (2005). Transforming growth factor-beta controls T helper type 1 cell development through regulation of natural killer cell interferon-gamma. Nat Immunol.

[34] Yu JH, Wei M, Becknell B, Trotta R, Liu SJ, Boyd Z, Jaung MS, Blaser BW, Sun J, Benson DM (2006). Pro- and antiinflammatory cytokine signaling: Reciprocal antagonism regulates interferon-gamma production by human natural killer cells. Immunity.

[35] Castriconi R, Cantoni C, Della Chiesa M, Vitale M, Marcenaro E, Conte R, Biassoni R, Bottino C, Moretta L, Moretta A (2003). Transforming growth factor beta 1 inhibits expression of NKp30 and NKG2D receptors: consequences for the NK-mediated killing of dendritic cells. Proc Natl Acad Sci USA.

[36] Crane CA, Han SJ, Barry JJ, Ahn BJ, Lanier LL, Parsa AT (2010). TGF-beta downregulates the activating receptor NKG2D on NK cells and CD8(+) T cells glioma patients. Neuro Oncol.

[37] Lee JC, Ahn YO, Kim DW, Heo DS (2004). Elevated TGF-b1 secretion and downmodulation of NKG2D underlies impaired NK cytotoxicity in cancer patients. J Immunother.

[38] Ramalingam R, Larmonier CB, Thurston RD, Midura-Kiela MT, Zheng SG, Ghishan FK, Kiela PR (2012). Dendritic cell-specific disruption of TGF-beta receptor II leads to altered regulatory T cell phenotype and spontaneous multiorgan autoimmunity. J Immunol.

[39] Dumitriu IE, Dunbar DR, Howie SE, Sethi T, Gregory CD (2009). Human dendritic cells produce TGF-beta 1 under the influence of lung carcinoma cells and prime the differentiation of CD4(+)CD25(+)Foxp3(+) regulatory T cells. J Immunol.

[40] Papaspyridonos M, Matei I, Huang YJ, Andre MD, Brazier-Mitouart H, Waite JC, Chan AS, Kalter J, Ramos I, Wu Q (2015). Id1 suppresses anti-tumour immune responses and promotes tumour progression by impairing myeloid cell maturation. Nat Commun.

[41] Belladonna ML, Volpi C, Bianchi R, Vacca C, Orabona C, Pallotta MT, Boon L, Gizzi S, Fioretti MC, Grohmann U (2008). Cutting edge: autocrine TGF-beta sustains default tolerogenesis by IDO-competent dendritic cells. J Immunol.

[42] Pallotta MT, Orabona C, Volpi C, Vacca C, Belladonna ML, Bianchi R, Servillo G, Brunacci C, Calvitti M, Bicciato S (2011). Indoleamine 2,3-dioxygenase is a signaling protein in long-term tolerance by dendritic cells. Nat Immunol.

[43] Kelly A, Gunaltay S, McEntee CP, Shuttleworth EE, Smedley C, Houston SA, Fenton TM, Levison S, Mann ER, Travis MA (2018). Human monocytes and macrophages regulate immune tolerance via integrin alpha v beta 8-mediated TGF beta activation. J Exp Med.

[44] Arwert EN, Harney AS, Entenberg D, Wang YR, Sahai E, Pollard JW, Condeelis JS (2018). A unidirectional transition from migratory to perivascular macrophage is required for tumor cell intravasation. Cell Rep.

[45] Lee YS, Park JS, Kim JH, Jung SM, Lee JY, Kim SJ, Park SH (2011). Smad6-specific recruitment of Smurf E3 ligases mediates TGF-beta 1-induced degradation of MyD88 in TLR4 signalling. Nat Commun.

[46] Hong S, Lim S, Li AG, Lee C, Lee YS, Lee EK, Park SH, Wang XJ, Kim SJ (2007). Smad7 binds to the adaptors TAB2 and TAB3 to block recruitment of the kinase TAK1 to the adaptor TRAF2. Nat Immunol.

[47] Pang Y, Gara SK, Achyut BR, Li Z, Yan HH, Day CP, Weiss JM, Trinchieri G, Morris JC, Yang L (2013). TGF-beta signaling in myeloid cells is required for tumor metastasis. Cancer Discov.

[48] Li ZY, Pang YL, Gara SK, Achyut BR, Heger C, Goldsmith PK, Lonning S, Yang L (2012). Gr-1+CD11b+cells are responsible for tumor promoting effect of TGF-ss in breast cancer progression. Int J Cancer.

[49] Jayaraman P, Parikh F, Newton JM, Hanoteau A, Rivas C, Krupar R, Rajapakshe K, Pathak R, Kanthaswamy K, MacLaren C (2018). TGF-beta 1 programmed myeloid-derived suppressor cells (MDSC) acquire immune-stimulating and tumor killing activity capable of rejecting established tumors in combination with radiotherapy. Oncoimmunology..

[50] Lee CR, Lee W, Cho SK, Park SG (2018). Characterization of multiple cytokine combinations and TGF-beta on differentiation and functions of myeloid-derived suppressor cells. Int J Mol Sci..

[51] Li Z, Zhang LJ, Zhang HR, Tian GF, Tian J, Mao XL, Jia ZH, Meng ZY, Zhao LQ, Yin ZN, Wu ZZ. Tumor-derived transforming growth factor-β is critical for tumor progression and evasion from immune surveillance. Asian Pac J Cancer Prev. 2014;15(13):5181–6. 10.7314/apjcp.2014.15.13.5181. 10.7314/apjcp.2014.15.13.5181 25040972

[52] Oh SA, Li MO (2013). TGF-β: guardian of T cell function. J Immunol.

[53] Tang N, Cheng C, Zhang X, Qiao M, Li N, Mu W, Wei XF, Han W, Wang H (2020). TGF-β inhibition via CRISPR promotes the long-term efficacy of CAR T cells against solid tumors. JCI Insight.

[54] Kloss CC, Lee J, Zhang A, Chen F, Melenhorst JJ, Lacey SF, Maus MV, Fraietta JA, Zhao Y, June CH (2018). Dominant-negative TGF-β receptor enhances PSMA-targeted human CAR T cell proliferation and augments prostate cancer eradication. Mol Ther..

[55] Friedman E, Gold LI, Klimstra D, Zeng ZS, Winawer S, Cohen A (1995). High-levels of transforming growth-factor-beta-1 correlate with disease progression in human colon cancer. Cancer Epidem Biomar.

[56] Lampropoulos P, Zizi-Sermpetzoglou A, Rizos S, Kostakis A, Nikiteas N, Papavassiliou AG (2012). TGF-beta signalling in colon carcinogenesis. Cancer Lett.

[57] Bierie B, Moses HL (2006). TGF beta: the molecular Jekyll and Hyde of cancer. Nat Rev Cancer.

[58] Shen W, Tao GQ, Zhang Y, Cai B, Sun J, Tian ZQ (2017). TGF-beta in pancreatic cancer initiation and progression: two sides of the same coin. Cell Biosci.

[59] Abou-Shady M, Baer HU, Friess H, Berberat P, Zimmermann A, Graber H, Gold LI, Korc M, Buchler MW (1999). Transforming growth factor betas and their signaling receptors in human hepatocellular carcinoma. Am J Surg.

[60] Lin TH, Shao YY, Chan SY, Huang CY, Hsu CH, Cheng AL (2015). High serum transforming growth factor-beta 1 levels predict outcome in hepatocellular carcinoma patients treated with sorafenib. Clin Cancer Res.

[61] Coskun HSI, Ozcan M, Ayaz S, Dalva K, Ustun C, Arat M (2006). Serum transforming growth factor beta 1 levels in multiple myeloma patients. Turk J Hematol.

[62] Watanabe Y, Iwamura A, Shimada YJ, Wakai K, Tamakoshi A, Iso H, Grp JS (2016). Transforming growth factor-beta 1 as a predictor for the development of hepatocellular carcinoma: a nested case-controlled study. Ebiomedicine.

[63] Havel JJ, Chowell D, Chan TA (2019). The evolving landscape of biomarkers for checkpoint inhibitor immunotherapy. Nat Rev Cancer.

[64] Bai X, Yi M, Jiao Y, Chu Q, Wu K (2019). Blocking TGF-beta signaling to enhance the efficacy of immune checkpoint inhibitor. Onco Targets Ther.

[65] Martin CJ, Datta A, Littlefield C, Kalra A, Chapron C, Wawersik S, Dagbay KB, Brueckner CT, Nikiforov A, Danehy FT (2020). Selective inhibition of TGF beta 1 activation overcomes primary resistance to checkpoint blockade therapy by altering tumor immune landscape. Sci Transl Med.

[66] Huynh LK, Hipolito CJ, Ten Dijke P (2019). A perspective on the development of TGF-beta inhibitors for cancer treatment. Biomolecules.

[67] Ciardiello D, Elez E, Tabernero J, Seoane J (2020). Clinical development of therapies targeting TGF beta: current knowledge and future perspectives. Ann Oncol.

[68] Jin CH, Krishnaiah M, Sreenu D, Subrahmanyam VB, Rao KS, Lee HJ, Park SJ, Park HJ, Lee K, Sheen YY (2014). Discovery of N-((4-([1,2,4]triazolo[1,5-a]pyridin-6-yl)-5-(6-methylpyridin-2-yl)-1H-imidazol-2 -yl)methyl)-2-fluoroaniline (EW-7197): a highly potent, selective, and orally bioavailable inhibitor of TGF-beta type I receptor kinase as cancer immunotherapeutic/antifibrotic agent. J Med Chem.

[69] Son JY, Park SY, Kim SJ, Lee SJ, Park SA, Kim MJ, Kim SW, Kim DK, Nam JS, Sheen YY (2014). EW-7197, a novel ALK-5 kinase inhibitor, potently inhibits breast to lung metastasis. Mol Cancer Ther.

[70] Jung SY, Hwang S, Clarke JM, Bauer TM, Keedy VL, Lee H, Park N, Kim SJ, Lee JI (2020). Pharmacokinetic characteristics of vactosertib, a new activin receptor-like kinase 5 inhibitor, in patients with advanced solid tumors in a first-in-human phase 1 study. Invest New Drugs.

[71] Jung SY, Yug JS, Clarke JM, Bauer TM, Keedy VL, Hwang S, Kim SJ, Chung EK, Lee JI (2020). Population pharmacokinetics of vactosertib, a new TGF-beta receptor type Iota inhibitor, in patients with advanced solid tumors. Cancer Chemother Pharmacol.

[72] Lee KW, Park YS, Ahn JB, Rha SY, Kim HK, Lee PY, Ryu MH, Lee J, Lee JK, Hwang S (2020). Safety and anti-tumor activity of the transforming growth factor beta receptor I kinase inhibitor, vactosertib, in combination with pembrolizumab in patients with metastatic colorectal or gastric cancer. J Immunother Cancer.

[73] Han JY, Pyo KH, Kim JH, Xin CF, Lee JK, Hwang S, Kim SJ, Cho BC, Cho BC (2020). Safety and anti-tumor activity of the transforming growth factor beta receptor I kinase inhibitor, vactosertib, in combination with durvalumab in patients with advanced non-small cell lung cancer (NSCLC). J Immunother Cancer.

[74] Malek E, Kim BG, Valent J, Driscoll J, Caimi P, Kim SJ, de Lima M, Letterio J (2018). Preclinical studies and a phase I trial of the tgf-beta receptor inhibitor, vactosertib (TEW-7197), in combination with pomalidomide in patients with multiple myeloma refractory to bortezomib or lenalidomide. Blood.

[75] Malek E, Hwang S, de Lima M, Caimi P, Gallogly MM, Metheny L, Otegbeye F, Tomlinson BK, Boughan KM, Cooper B et al. Preclinical studies and phase i trial of vactosertib in combination with pomalidomide in relapsed multiple myeloma: A corticosteroid-free approach by targeting TGF-β signaling pathway. Blood. 2019;134(Supplement 1):3232.

[76] Bataller A, Montalban-Bravo G, Soltysiak KA, Garcia-Manero G (2019). The role of TGFbeta in hematopoiesis and myeloid disorders. Leukemia.

[77] Aluri S, Bachiashvili K, Budhathoki A, Bhagat TD, Choudhary GS, Gordon S, Ramachandra N, Pradhan K, Maqbool S, Shastri A, et al. Clinical ALK5 inhibitor, vactosertib, reverses TGFβ-1 stimulated Smad-2 driven ineffective hematopoiesis in MDS. Blood. 2019;134 (Suppl. 1):2990.

[78] Tsukada T, Fushida S, Harada S, Terai S, Yagi Y, Kinoshita J, Oyama K, Tajima H, Ninomiya I, Fujimura T (2013). Low-dose paclitaxel modulates tumour fibrosis in gastric cancer. Int J Oncol.

[79] Fujiwara Y, Nokihara H, Yamada Y, Yamamoto N, Sunami K, Utsumi H, Asou H, Takahash IO, Ogasawara K, Gueorguieva I (2015). Phase 1 study of galunisertib, a TGF-beta receptor I kinase inhibitor, in Japanese patients with advanced solid tumors. Cancer Chemother Pharmacol.

[80] Rodon J, Carducci M, Sepulveda-Sanchez JM, Azaro A, Calvo E, Seoane J, Brana I, Sicart E, Gueorguieva I, Cleverly A (2015). Pharmacokinetic, pharmacodynamic and biomarker evaluation of transforming growth factor-beta receptor I kinase inhibitor, galunisertib, in phase 1 study in patients with advanced cancer. Invest New Drugs.

[81] Herbertz S, Sawyer JS, Stauber AJ, Gueorguieva I, Driscoll KE, Estrem ST, Cleverly AL, Desaiah D, Guba SC, Benhadji KA (2015). Clinical development of galunisertib (LY2157299 monohydrate), a small molecule inhibitor of transforming growth factor-beta signaling pathway. Drug Des Devel Ther.

[82] Faivre SJ, Santoro A, Gane E, Kelley RK, Hourmand II, Assenat E, Gueorguieva I, Cleverly A, Desaiah D, Lahn MMF (2016). A phase 2 study of galunisertib, a novel transforming growth factor-beta (TGF-beta) receptor I kinase inhibitor in patients with advanced hepatocellular carcinoma (HCC) and low serum alpha fetoprotein (AFP). J Clin Oncol.

[83] Gueorguieva I, Cleverly AL, Stauber A, Sada Pillay N, Rodon JA, Miles CP, Yingling JM, Lahn MM (2014). Defining a therapeutic window for the novel TGF-beta inhibitor LY2157299 monohydrate based on a pharmacokinetic/pharmacodynamic model. Br J Clin Pharmacol.

[84] Kovacs RJ, Maldonado G, Azaro A, Fernandez MS, Romero FL, Sepulveda-Sanchez JM, Corretti M, Carducci M, Dolan M, Gueorguieva I (2015). Cardiac safety of TGF-beta receptor I kinase inhibitor LY2157299 monohydrate in cancer patients in a first-in-human dose study. Cardiovasc Toxicol.

[85] Parente P, Parcesepe P, Covelli C, Olivieri N, Remo A, Pancione M, Latiano TP, Graziano P, Maiello E, Giordano G. Crosstalk between the tumor microenvironment and immune system in pancreatic ductal adenocarcinoma: potential targets for new therapeutic approaches. Gastroenterol Res Pract*.* 2018; 7530619. 10.1155/2018/7530619 PMC6312626 30662458

[86] Melisi D, Garcia-Carbonero R, Macarulla T, Pezet D, Deplanque G, Fuchs M, Trojan J, Oettle H, Kozloff M, Cleverly A (2016). A phase II, double-blind study of galunisertib plus gemcitabine (GG) vs gemcitabine plus placebo (GP) in patients (pts) with unresectable pancreatic cancer (PC). J Clin Oncol.

[87] Pei HX, Parthasarathy S, Joseph S, McMillen W, Xu XH, Castaneda S, Inigo I, Britt K, Anderson B, Zhao GY (2017). LY3200882, a novel, highly selective TGF beta RI small molecule inhibitor. Cancer Res.

[88] Yap TBC, Massard C. First-in-human phase 1 dose-escalation trial of the potent and selective next generation transforming growth factor-&beta; receptor type 1 (TGF-&beta;R1) inhibitor LY3200882 in patients with advanced cancer. In Proceedings from the 2018 SITC annual meeting. 2018; Abstract 030.

[89] Gordon MS, Ilaria R, de Alwis DP, Mendelson DS, McKane S, Wagner MM, Look KY, LoRusso PM (2013). A phase I study of tasisulam sodium (LY573636 sodium), a novel anticancer compound, administered as a 24-h continuous infusion in patients with advanced solid tumors. Cancer Chemother Pharmacol.

[90] Tojo M, Hamashima Y, Hanyu A, Kajimoto T, Saitoh M, Miyazono K, Node M, Imamura T (2005). The ALK-5 inhibitor A-83-01 inhibits Smad signaling and epithelial-to-mesenchymal transition by transforming growth factor-beta. Cancer Sci.

[91] Sim WJ, Iyengar PV, Lama D, Lui SKL, Ng HC, Haviv-Shapira L, Domany E, Kappei D, Tan TZ, Saei A (2019). c-Met activation leads to the establishment of a TGF beta-receptor regulatory network in bladder cancer progression. Nat Commun.

[92] Melisi D, Ishiyama S, Sclabas GM, Fleming JB, Xia Q, Tortora G, Abbruzzese JL, Chiao PJ (2008). LY2109761, a novel transforming growth factor beta receptor type I and type II dual inhibitor, as a therapeutic approach to suppressing pancreatic cancer metastasis. Mol Cancer Ther.

[93] Connolly EC, Saunier EF, Quigley D, Luu MT, De Sapio A, Hann B, Yingling JM, Akhurst RJ (2011). Outgrowth of drug-resistant carcinomas expressing markers of tumor aggression after long-term TbetaRI/II kinase inhibition with LY2109761. Cancer Res.

[94] Bandyopadhyay A, Agyin JK, Wang L, Tang YP, Lei XF, Story BM, Cornell JE, Pollock BH, Mundy GR, Sun LZ (2006). Inhibition of pulmonary and skeletal metastasis by a TGF-beta type I receptor kinase inhibitor. Can Res.

[95] Ehata S, Hanyu A, Fujime M, Katsuno Y, Fukunaga E, Goto K, Ishikawa Y, Nomura K, Yokoo H, Shimizu T (2007). Ki26894, a novel transforming growth factor-beta type I receptor kinase inhibitor, inhibits in vitro invasion and in vivo bone metastasis of a human breast cancer cell line. Cancer Sci.

[96] Shinto O, Yashiro M, Kawajiri H, Shimizu K, Shimizu T, Miwa A, Hirakawa K (2010). Inhibitory effect of a TGFbeta receptor type-I inhibitor, Ki26894, on invasiveness of scirrhous gastric cancer cells. Br J Cancer.

[97] Byfield SD, Roberts AB. Lateral signaling enhances TGF-beta response complexity. Trends Cell Biol. 2004; 14(3):107–11. 10.1016/j.tcb.2004.01.001 15055198

[98] Sawyer TK (2004). Novel oncogenic protein kinase inhibitors for cancer therapy. Curr Med Chem Anticancer Agents.

[99] Matsuyama S, Iwadate M, Kondo M, Saitoh M, Hanyu A, Shimizu K, Aburatani H, Mishima HK, Imamura T, Miyazono K (2003). SB-431542 and Gleevec inhibit transforming growth factor-beta-induced proliferation of human osteosarcoma cells. Cancer Res.

[100] Takeuchi K, Abe M, Hiasa M, Oda A, Amou H, Kido S, Harada T, Tanaka O, Miki H, Nakamura S (2010). Tgf-Beta inhibition restores terminal osteoblast differentiation to suppress myeloma growth. PLoS ONE.

[101] Uhl M, Aulwurm S, Wischhusen J, Weiler M, Ma JY, Almirez R, Mangadu R, Liu YW, Platten M, Herrlinger U (2004). SD-208, a novel transforming growth factor beta receptor I kinase inhibitor, inhibits growth and invasiveness and enhances immunogenicity of murine and human glioma cells in vitro and in vivo. Cancer Res.

[102] Mohammad KS, Javelaud D, Fournier PG, Niewolna M, McKenna CR, Peng XH, Duong V, Dunn LK, Mauviel A, Guise TA (2011). TGF-beta-RI kinase inhibitor SD-208 reduces the development and progression of melanoma bone metastases. Cancer Res.

[103] Subramanian G, Schwarz RE, Higgins L, McEnroe G, Chakravarty S, Dugar S, Reiss M (2004). Targeting endogenous transforming growth factor beta receptor signaling in SMAD4-deficient human pancreatic carcinoma cells inhibits their invasive phenotype1. Cancer Res.

[104] Lee GT, Hong JH, Mueller TJ, Watson JA, Kwak C, Sheen YY, Kim DK, Kim SJ, Kim IY (2008). Effect of IN-1130, a small molecule inhibitor of transforming growth factor-beta type I receptor/activin receptor-like kinase-5, on prostate cancer cells. J Urol.

[105] Park CY, Min KN, Son JY, Park SY, Nam JS, Kim DK, Sheen YY (2014). An novel inhibitor of TGF-beta type I receptor, IN-1130, blocks breast cancer lung metastasis through inhibition of epithelial-mesenchymal transition. Cancer Lett.

[106] Morris JC, Tan AR, Olencki TE, Shapiro GI, Dezube BJ, Reiss M, Hsu FJ, Berzofsky JA, Lawrence DP (2014). Phase I study of GC1008 (fresolimumab): a human anti-transforming growth factor-beta (TGFbeta) monoclonal antibody in patients with advanced malignant melanoma or renal cell carcinoma. PLoS ONE.

[107] Tolcher AW, Berlin JD, Cosaert J, Kauh J, Chan E, Piha-Paul SA, Amaya A, Tang S, Driscoll K, Kimbung R (2017). A phase 1 study of anti-TGFbeta receptor type-II monoclonal antibody LY3022859 in patients with advanced solid tumors. Cancer Chemother Pharmacol.

[108] Fjellbirkeland L, Cambier S, Broaddus VC, Hill A, Brunetta P, Dolganov G, Jablons D, Nishimura SL (2003). Integrin alphavbeta8-mediated activation of transforming growth factor-beta inhibits human airway epithelial proliferation in intact bronchial tissue. Am J Pathol.

[109] Eberlein C, Kendrew J, McDaid K, Alfred A, Kang JS, Jacobs VN, Ross SJ, Rooney C, Smith NR, Rinkenberger J (2013). A human monoclonal antibody 264RAD targeting alphavbeta6 integrin reduces tumour growth and metastasis, and modulates key biomarkers in vivo. Oncogene.

[110] Moore KM, Thomas GJ, Duffy SW, Warwick J, Gabe R, Chou P, Ellis IO, Green AR, Haider S, Brouilette K, et al. Therapeutic targeting of integrin alphavbeta6 in breast cancer. J Natl Cancer Inst. 2014;106(8). 10.1093/jnci/dju169 PMC4151855 24974129

[111] Nam JS, Terabe M, Mamura M, Kang MJ, Chae H, Stuelten C, Kohn E, Tang B, Sabzevari H, Anver MR (2008). An anti-transforming growth factor beta antibody suppresses metastasis via cooperative effects on multiple cell compartments. Cancer Res.

[112] Arteaga CL, Hurd SD, Winnier AR, Johnson MD, Fendly BM, Forbes JT. Anti-transforming growth factor (TGF)-beta antibodies inhibit breast cancer cell tumorigenicity and increase mouse spleen natural killer cell activity. Implications for a possible role of tumor cell/host TGF-beta interactions in human breast cancer progression. J Clin Invest. 1993; 92(6):2569–76. 10.1172/JCI116871 PMC288452 7504687

[113] Ganapathy V, Ge R, Grazioli A, Xie W, Banach-Petrosky W, Kang Y, Lonning S, McPherson J, Yingling JM, Biswas S (2010). Targeting the transforming growth factor-beta pathway inhibits human basal-like breast cancer metastasis. Mol Cancer..

[114] O'Connor-McCourt MD, Tremblay G, Lenferink A, Sulea T, Zwaagstra J, Koropatnick J (2018). AVID200, a highly potent TGF-beta trap, exhibits optimal isoform selectivity for enhancing anti-tumor T-cell activity, without promoting metastasis or cardiotoxicity. Cancer Res.

[115] Strauss J, Heery CR, Schlom J, Madan RA, Cao L, Kang ZG, Lamping E, Marte JL, Donahue RN, Grenga I (2018). Phase I trial of M7824 (MSB0011359C), a bifunctional fusion protein targeting PD-L1 and TGF beta. Adv Solid Tumors Clin Cancer Res.

[116] Paz-Ares L, Kim TM, Vicente D, Felip E, Lee DH, Lee KH, Lin CC, Flor MJ, Di Nicola M, Alvarez RM (2020). Bintrafusp Alfa, a bifunctional fusion protein targeting TGF-beta and PD-L1, in second-line treatment of patients with NSCLC: results from an expansion cohort of a phase 1 trial. J Thorac Oncol.

[117] Birrer MJ, Mileshkin LR, Fujiwara K, Ray-Coquard I, Alexandre J, Okamoto A, Mirza MR, Gulley JL, Jehl G, Ramage S (2020). Phase II study of bintrafusp alfa, a bifunctional fusion protein targeting TGF-beta and PD-L1, in platinum-experienced advanced cervical cancer. Ann Oncol..

[118] Fenaux P, Platzbecker U, Mufti GJ, Garcia-Manero G, Buckstein R, Santini V, Diez-Campelo M, Finelli C, Cazzola M, Ilhan O (2020). Luspatercept in patients with lower-risk myelodysplastic syndromes. New Engl J Med.

[119] Bandyopadhyay A, Lopez-Casillas F, Malik SN, Montiel JL, Mendoza V, Yang J, Sun LZ (2002). Antitumor activity of a recombinant soluble betaglycan in human breast cancer xenograft. Cancer Res.

[120] Muraoka RS, Dumont N, Ritter CA, Dugger TC, Brantley DM, Chen J, Easterly E, Roebuck LR, Ryan S, Gotwals PJ (2002). Blockade of TGF-beta inhibits mammary tumor cell viability, migration, and metastases. J Clin Invest.

[121] Gleave ME, Monia BP (2005). Antisense therapy for cancer. Nat Rev Cancer.

[122] Dias N, Stein CA (2002). Antisense oligonucleotides: basic concepts and mechanisms. Mol Cancer Ther.

[123] Tibbitt MW, Dahlman JE, Langer R (2016). Emerging frontiers in drug delivery. J Am Chem Soc.

[124] Schlingensiepen KH, Schlingensiepen R, Steinbrecher A, Hau P, Bogdahn U, Fischer-Blass B, Jachimczak P (2006). Targeted tumor therapy with the TGF-beta 2 antisense compound AP 12009. Cytokine Growth Factor Rev.

[125] Jaschinski F, Rothhammer T, Jachimczak P, Seitz C, Schneider A, Schlingensiepen KH (2011). The antisense oligonucleotide trabedersen (AP 12009) for the targeted inhibition of TGF-beta2. Curr Pharm Biotechnol.

[126] Hau P, Jachimczak P, Bogdahn U (2009). Treatment of malignant gliomas with TGF-beta2 antisense oligonucleotides. Expert Rev Anticancer Ther.

[127] Oh J, Barve M, Matthews CM, Koon EC, Heffernan TP, Fine B, Grosen E, Bergman MK, Fleming EL, DeMars LR (2016). Phase II study of Vigil DNA engineered immunotherapy as maintenance in advanced stage ovarian cancer. Gynecol Oncol.

[128] Tong AW (2006). Small RNAs and non-small cell lung cancer. Curr Mol Med.

[129] Giaccone G, Bazhenova LA, Nemunaitis J, Tan M, Juhasz E, Ramlau R, van den Heuvel MM, Lal R, Kloecker GH, Eaton KD (2015). A phase III study of belagenpumatucel-L, an allogeneic tumour cell vaccine, as maintenance therapy for non-small cell lung cancer. Eur J Cancer.

[130] Jakowlew SB, Mathias A, Chung P, Moody TW (1995). Expression of transforming growth factor beta ligand and receptor messenger RNAs in lung cancer cell lines. Cell Growth Differ.

[131] Constam DB, Philipp J, Malipiero UV, ten Dijke P, Schachner M, Fontana A (1992). Differential expression of transforming growth factor-beta 1, -beta 2, and -beta 3 by glioblastoma cells, astrocytes, and microglia. J Immunol.

[132] Nemunaitis J, Dillman RO, Schwarzenberger PO, Senzer N, Cunningham C, Cutler J, Tong A, Kumar P, Pappen B, Hamilton C (2006). Phase II study of belagenpumatucel-L, a transforming growth factor beta-2 antisense gene-modified allogeneic tumor cell vaccine in non-small-cell lung cancer. J Clin Oncol.

[133] Rook AH, Kehrl JH, Wakefield LM, Roberts AB, Sporn MB, Burlington DB, Lane HC, Fauci AS (1986). Effects of transforming growth factor beta on the functions of natural killer cells: depressed cytolytic activity and blunting of interferon responsiveness. J Immunol.

[134] Tsunawaki S, Sporn M, Ding A, Nathan C (1988). Deactivation of macrophages by transforming growth factor-beta. Nature.

